# MUC 15 Promotes Osteosarcoma Cell Proliferation, Migration and Invasion through Livin, MMP-2/MMP-9 and Wnt/β-Catenin Signal Pathway

**DOI:** 10.7150/jca.49641

**Published:** 2021-01-01

**Authors:** Tonglei Chen, Zhenshi Chen, Xiaoning Lian, Weidong Wu, Lei Chu, Shaoru Zhang, Lihui Wang

**Affiliations:** 1 Suzhou Ninth People's Hospital, Suzhou, Jiangsu 215200, China.; 2 Danyang People's Hospital of Jiangsu Province, Danyang, Jiangsu 212300, China.; 3 Danyang Hospital Affiliated to Nantong University, Danyang, Jiangsu 212300, China.; 4 Department of Assay Development, TOT BIOPHARM co., LTD, Suzhou, Jiangsu 215024, China.

**Keywords:** Osteosarcoma, MUC 15, proliferation, migration, invasion

## Abstract

**Objective:** To investigate the high expression of MUC15 in promoting proliferation, migration and invasion in osteosarcoma (OS) cell and its potential mechanism.

**Methods:** The expressions of MUC15 in OS patients were analyzed from GEO Datasets, tumor cell lines and clinical samples. The roles of MUC15 in OS were explored by CCK-8, flow cytometry, transwell and western blot assay, respectively.

**Results:** MUC15 was highly expressed in osteosarcoma, and there was a significant negative correlation between MUC15 and the prognosis. Knockdown of MUC15 in HOS and U-2OS could promote tumor cell apoptosis, down-regulate the expression of MMP2/9, reduce the epithelial interstitial transition and silence the Wnt/b-Catenin signal pathway.

**Conclusion:** The high-expression of MUC15 promotes the proliferation, migration and invasion of osteosarcoma through anti-apoptosis, increasing the invasive ability by epithelial interstitial transition, and activating the Wnt/b-Catenin signal pathway.

## Introduction

Osteosarcoma (OS) is the most common primary bone malignancy, which has the highest incidence in children and adolescents. As a bone tumor with high malignant degree and poor prognosis, it seriously threats the physical and mental health of patients 1. With an understanding of the biological characteristics of bone tumor, the improvement of surgical techniques, the establishment of clinical staging system, the use of new adjuvant chemotherapy and the development of imaging technology in the treatment process, the 5 year survival rate of OS increased from 20% to 65%-70% 2, 3. However, in the past two decades, there has been no further improvement in survival. Among the newly diagnosed patients with OS, about 20% of the patients have distant metastasis, and 90% of them are pulmonary metastasis. Once OS has metastasis and recurrence, even with new adjuvant chemotherapy, the 5 year survival rate of these patients is only 20% to 30% 4-6.

The recurrence and metastasis are main problems restricting the therapeutic effect of OS 7. In view of the poor prognosis of OS, some researchers tried to explore the changes of its gene and molecular mechanisms through individual diagnosis, such as gene sequencing and chemotherapeutic drug screening. Great progress has been reported that chromosome abnormality, tumor suppressor gene abnormality, transcription factor, growth factor, WWOX and miRNAs play important roles in the occurrence and development of OS 8-16. However, the above research is still in its infancy in use, and there is no sufficient evidence to show that patients' benefit from it. At present, the etiology and pathogenesis of OS have not been fully elucidated 17. Hence, it is necessary to do further understand the biological characteristics and pathogenesis of OS to promote the development of targeted therapy for primary and metastatic OS.

Mucin (MUC) family is a kind of highly glycosylated proteins, which mainly provides lubrication and protective chemical barrier functions. In cancer research, MUC has many other special functions, involving tumor cell proliferation, apoptosis, migration, adhesion, invasion and drug resistance. MUC15, a cell transmembrane mucin, has been reported over-expressed in papillary thyroid carcinoma and colorectal carcinomas, which was negative to the prognosis 18, 19. The potential role of MUC15 in OS has never been reported.

In this manuscript, we focused on the effects and molecular mechanisms of MUC15 that contribute to the progression and metastasis of OS. As a potential therapeutic target, MUC15 will have major implications for the treatment of OS.

## Materials and methods

### Cell lines

The normal human osteoblast hFOB1.19 cell line and the human Osteosarcoma HOS, MG63 and U-2OS cell lines were purchased from Shanghai Institute of Biochemistry and Cell Biology (SIBCB). The hFOB1.19 cell line was cultured at 34℃ and the human OS cell lines were cultured at 37℃ in 5% CO_2_ cell culture incubator. MUC15 knockdown (MUC15 KD) and negative control (MUC15 NC) of HOS and U-2OS cell lines were established by using lentivirus expressing MUC15-targeting (5'-GATCCCCACAGCCACGGAATAACAGATTCAAGAGATCTGTTATTCCGTGGCTGTTTTTA-3') and scrambled control (5'-GATCCCCTTCTCCGAACGTGTCACGTTTCAAGAGAACGTGA- CACGTTCGGAGAATTTTTA-3') shRNA (Hanbio Biotechnoloy, Shanghai, China) respectively.

### Reagents and media

Cells were cultured in DMEM medium supplemented with 10% heat-inactivated fetal bovine serum (FBS), 100 μg/mL streptomycin and 100 IU/mL penicillin (Gibco). G418 and cisplatin (DDP) were purchased from Sigma.

### Patients and clinical samples

This study enrolled 41 patients underwent surgery and diagnosed to be confirmed OS in Suzhou Ninth People's Hospital and the People's Hospital of Danyang. Without preoperative chemotherapy or radiotherapy treatment, the OS tissues and corresponding adjacent normal tissues were kept frozen in a refrigerator.

### Immunohistochemistry

The tumor tissues of 41 osteosarcoma patients with complete follow-up information and normal bone tissues as controls were paraffin-embedded and MUC15 (Abcam) was located. The positive rate is generally divided as: 0 (0%), 1 (1-10%), 2 (11-50%), and 3 (>50%). The staining intensity has semiquantitatively been scored as: 0 (negative, -), 1 (weak, +), 2 (medium, ++), 3 (strong, +++). Then calculate the value of Immunoreactive score (IRS = positive rate × intensity). 10 HPF should be observed randomly. Three people are required to score independently 20. Taking the median of IRS as the threshold value, patients were divided into MUC15 high-expression group (IRS ≥ 4.3, n=22) and MUC15 low-expression group (IRS < 4.3, n=19).

### Western blot

The cells were prepared using RIPA lysis buffer as well as protease and phosphatase inhibitor (Bimake). 30mg of protein loaded per well and immune blotted overnight at 4 °C with antibody. The primary antibodies included anti-MUC15 (Abcam, ab224468), anti-Livin (Abcam, ab97350), anti-MMP2 (Invitrogen, 35-1300Z), anti-MMP9 (Invitrogen, MA5-15886), anti-E-cadherin (GeneTex, GTX124198), anti-Vimentin (Abcam, ab92547), anti-b-Catenin (CST, #9562) and anti-c-Myc (CST, #5605). The secondary antibodies included goat anti-mouse IgG HRP and goat anti-rabbit IgG HRP (Invitrogen).

### Assessment of tumor cell apoptosis

Apoptosis were quantified using Annexin V-FITC (Miltenyi, 130-092-052) to detect externalized phosphatidylserine and PI (Miltenyi, 130-092-052) to detect plasma membrane disruption. Tumor cells were firstly pretreated with DDP (10 mg/mL) for 24 h, and then assessed with Annexin V and PI in binding buffer for 15 min in the dark. Cells were collected using flow cytometer (BD Calibur) and the data were analyzed using FlowJo software.

### Cell proliferation assay

Cell Counting Kit-8 (CCK-8) was used (MCE) according to the manufacturer's protocol, and cell proliferation was determinate by colorimetric assays.

### Transwell assays

Cell invasion assay was performed by 8μm pore membranes Transwell plates (Corning) with Matrigel. 4 × 10^4^ cells were planted into the upper chambers with serum free medium. The lower chamber was offered with medium containing 10% FBS as chemoattractant. After 48 hours incubating, cells in the upper chambers were wiped by a brush. Then the membrane was stained with 0.1% crystal violet and noted by an inverted microscope. The migration ability of cells was detected by transwell chamber without Matrigel, and the method of detection was the same as that of invasion test.

### Wound healing assay

Cells were seeded in 6-well plates until allowed to reach confluence. Then each well was scraped to create a linear region devoid of cells with a pipette tip (10 μL). Cells cultured with DMEM medium (without FBS) for 24 hours. Then the healing of scratches was observed under microscope.

### Statistical analysis

Each experiment in this study was repeated for 3 times independently. Data were expressed as Mean ± SEM. Statistically significance of differences was performed using GraphPad Pad Prism 5 and SPSS 22.0 by Student's *t* test and ANOVA. For survival assays, comparisons were analyzed by a Log-rank test. *p*<0.05 was deemed as statistically significant.

## Results

### GEO Datasets and clinical specimens analyze the high expression of MUC15 in osteosarcoma

**Figure 1:**
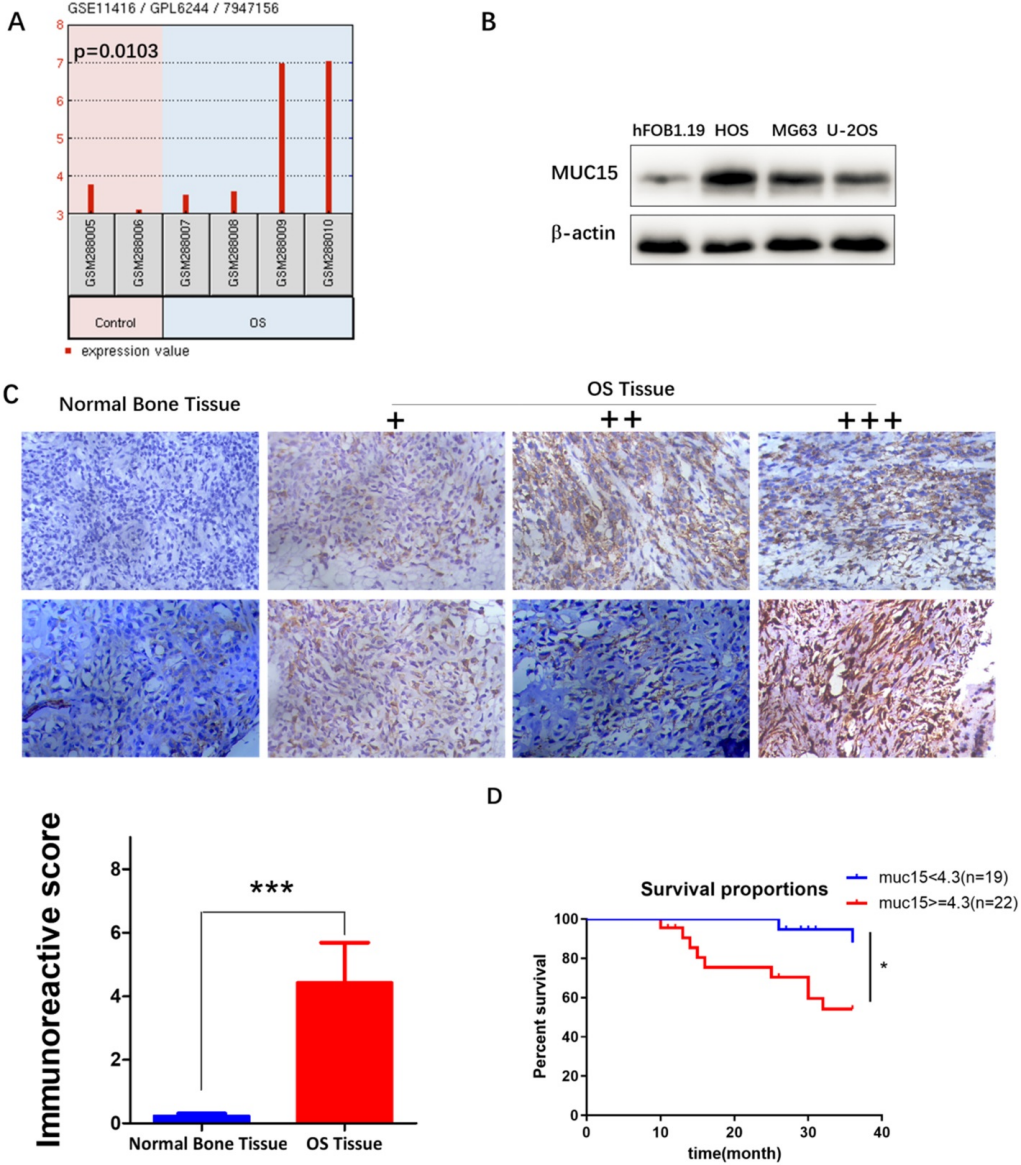
** MUC15 was highly expressed in OS. A:** Differential expressions of MUC15 in OS tissues and normal bone tissues were analyzed using the GEO Datasets (GSE11416 chip). **B:** The expressions of MUC15 in normal human osteoblast hFOB1.19 and human OS cell lines were detected by western blot. **C:** The expressions of MUC15 in OS tissues and normal bone tissues were detected by the immunohistochemical method. **D:** Survival differences of MUC15 in 41 patients with OS. The threshold value of 4.3 for high expression and low expression was referred to the median of IRS. *p < 0.05, *** *p*<0.001.

We first analyzed the expression of different gene between OS and normal bone tissues in GSE11416 chip form the GEO Datasets, and then screened the mRNA with more than 2-fold change of expression. MUC15 was found expressed significantly high in osteosarcoma tissues (*p*=0.0103, Fig. 1A). Then we further detected MUC15 expression in OS tumor cell lines and clinical samples to verify the results (Fig. 1B & C). At last, we evaluated the effect of MUC15 expression on the survival of patients with OS. The clinical data of 41 OS patients showed that MUC15 was significantly negatively to the survival of OS patients (*p*=0.0169, Fig. 1D, Fig. S1). These results demonstrated MUC15 expressed significant high in OS, which may be correlated with the poor prognosis.

### High-expression of MUC15 promotes proliferation, migration and invasion in OS cell

**Figure 2:**
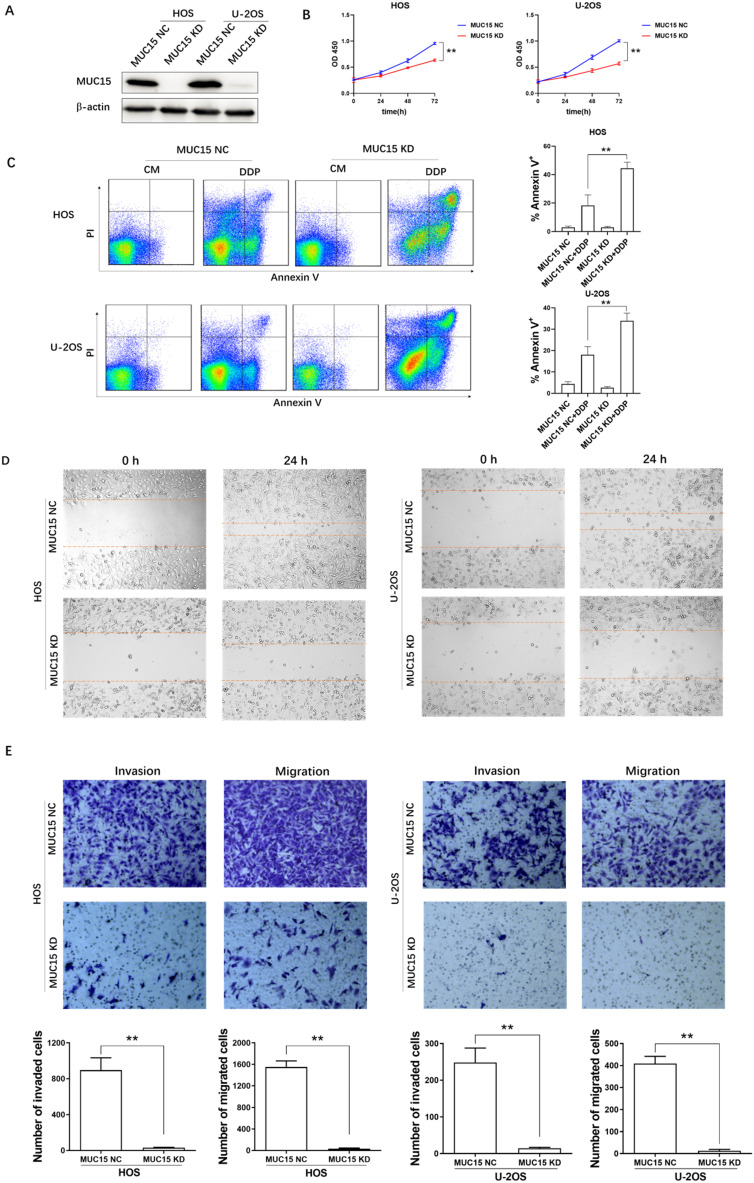
** The effects of high-expressed MUC15 in OS cells. A:** Western blot detected the knockdown of MUC15 in HOS and U-2OS cell lines. **B:** The proliferation of OS cells was detected by CCK-8. **C:** Flow cytometry analysis and quantification of apoptosis in the cells treated with DDP. **D, E:** The migration and invasion of HOS and U-2OS were detected by wound healing assay and transwell assay. Data (mean ± SEM) represented three independent experiments, **p<0.01.

Proliferation, migration and invasion are the key factors of tumor malignant transformation. To investigate the potential effect of MUC15 in OS cell, we knocked down the expression of MUC15 in HOS and U-2OS cell lines by using RNA interference technology (Fig. 2A). At the cell line level, the effect of MUC15 expression in HOS and U-2OS proliferation, migration and invasion were detected by using CCK-8, flow cytometry, transwell and wound healing assay. The results showed that knockdown MUC15 of OS cells reduced the proliferation ratio, increased the apoptosis rate, and inhibited migration and invasion significantly (Fig. 2B-E). In summary, these data demonstrated that the high-expression of MUC15 plays a critical role in OS cells proliferation, migration and invasion and may be an important target for clinical treatment of OS.

### Mechanism of MUC15 on Osteosarcoma proliferation, migration and invasion

**Figure 3:**
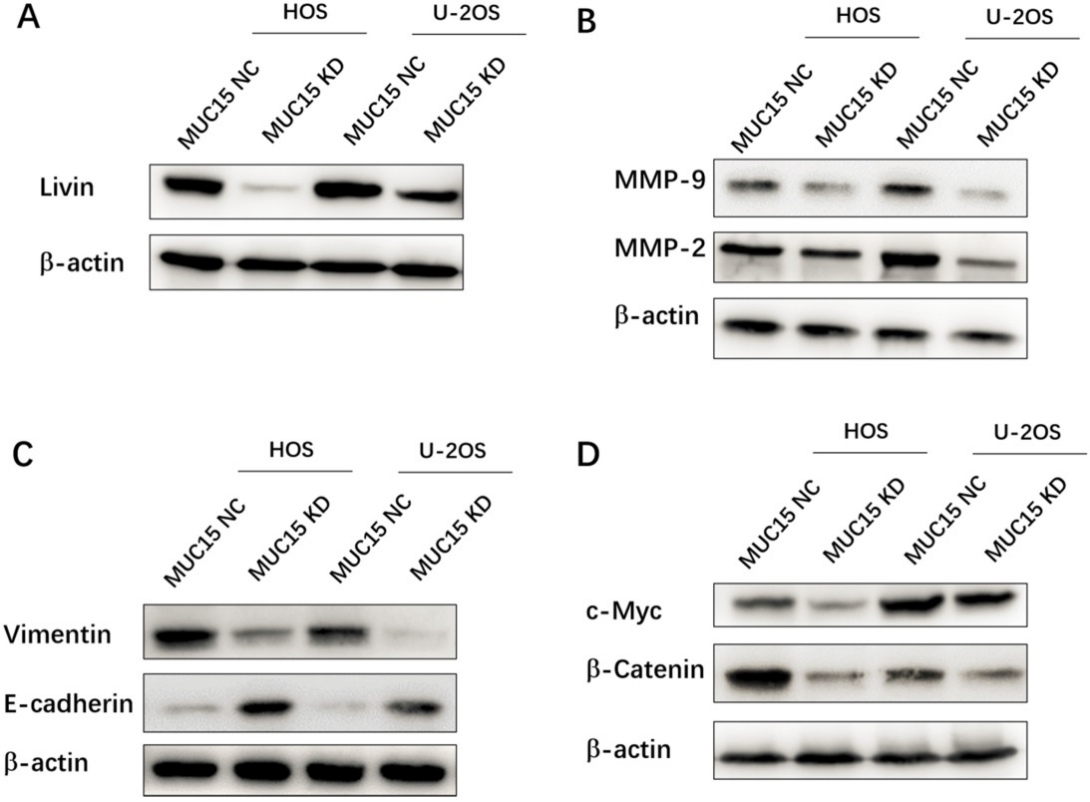
** Mechanisms of MUC15 on osteosarcoma proliferation, migration and invasion.** Western blot detected the apoptosis-inhibiting protein Livin (**A**), the migration-related proteins MMP-9 /MMP-2 (**B**), EMT related proteins (**C**) and the Wnt/b-Catenin signaling pathway of OS cell (**D**).

In this study, the possible mechanisms of MUC15 in progression and metastasis of OS were discussed. First of all, the different-expression of the apoptosis-inhibiting protein Livin in MUC15 NC (high) and MUC15 KD (low) OS cell lines suggested MUC15 affecting cell proliferation and apoptosis through Livin (Fig. 3A). Secondly, as the depth of invasion, metastatic distance and vascular permeability of OS cells are related to the expression of MMPs and EMT related proteins 21, 22. Compared with MUC15 NC cells, MMP-9, MMP-2 and Vimentin proteins in MUC15 KD group was significantly down-regulated, while E-cadherin was up-regulated, which partly explained how MUC15 promotes the migration and invasion of OS cells (Fig. 3B & C). Lastly, activation of signal pathways is often abnormal in the occurrence and development of OS. Hence, we detected the Wnt/β-Catenin signaling pathway which was important in regulating the biological characteristics of tumor cells and the progression of the disease 23. Western blot showed that the expression levels of b-Catenin and c-Myc (Wnt/b-Catenin signaling pathway-related proteins) in MUC15 KD OS cells were lower than that in MUC15 NC cells (Fig. 3D). These results suggested that MUC15 could promote OS cell proliferation, invasion and migration through resisting apoptosis, regulating the levels of MMPs and EMT related proteins, and activating Wnt/β-Catenin signal pathway.

## Discussion

In recent years, with great progress of research and a series of new breakthroughs have been made in the treatment of OS, limb salvage therapy supported by new adjuvant chemotherapy has been widely carried out in clinic, while life quality of patients has also been greatly improved. However, for the shortage understanding of the pathogenesis of OS, the survival and the prognosis of this invasive bone tumor has hardly improved. Patients with recurrent or metastatic osteosarcoma are usually resistant to standard chemotherapy. When traditional surgery and chemotherapy can no longer effectively control metastasis, new treatment strategies are urgent to be explored 24-26.

With the development of molecular biology research, researches on immunotherapy, gene therapy and molecular targeted therapy provide more hopes for the treatment of OS. Of note, molecular targeted therapy has stronger accuracy, specificity, and fewer side effects. In this regard, numerous studies have worked on searching for new therapeutic targets. Recently, molecules involved in OS cell migration, invasion, angiogenesis, apoptosis, and proliferation have been demonstrated as reliable biomarkers and therapeutic targets, such as IGF-R, EGFR, VEGF, AURKA, and some miRNAs/lncRNAs 27-29. Further research on the molecular targets and their mechanisms of osteosarcoma will hopefully provide new insights into the therapies.

MUC, as well-known solid tumor antigens (especially MUC16) are routinely used for monitoring disease. However, there is still little functional information of other transmembrane MUC. In this study, we first demonstrated the high-expression of MUC15 in OS tumor cell lines and clinical samples. Subsequently, we investigated the role and mechanism of MUC15 in promoting OS proliferation, migration and invasion, although a recent study of MUC15 reported a tumor suppressing role in renal cell carcinoma. MUC15 also has been shown as an oncogene in the development of cancer and influence cellular growth, adhesion, invasion, metastasis and immunosuppression. These evidences indicate that it plays different roles in different types of cancers, which may because MUC15 involved in lots of biological functional regulations.

Limitations of this research should not be ignored. Since antibodies were only specific to human, experiments could only be carried out at the cellular level. Molecular mechanism of MUC15 plays *in vivo* remains to be further confirmed. In addition, the relatively small sample size utilized likely contributes to the lack of significance in analyzing correlation with MUC15 expression and clinical characteristics (such as Enneking stage, age, gender, etc.). Future studies should incorporate more OS cell lines and evaluate more clinical cases. Targeted therapy strategies of MUC15 and its underlying mechanisms will provide a theoretical basis for the innovative therapies of OS with a bright future.

## Supplementary Material

Supplementary figures and tables.

## Abbreviations

CCK-8: Cell counting kit-8
EMT: Epithelial interstitial transition
FBS: Fetal bovine serum
IRS: Immunoreactive score
MMP: Matrix metalloproteinase
MUC: Mucin
OS: Osteosarcoma
