# Supplementary material for: MUC 15 Promotes Osteosarcoma Cell Proliferation, Migration and Invasion through Livin, MMP-2/MMP-9 and Wnt/β-Catenin Signal Pathway
Source: J Cancer. 2021 Jan 1;12(2):467–73. doi: 10.7150/jca.49641 (PMC7739004; doi:10.7150/jca.49641)
Supplement: Supplementary file 1 — Supplementary figures and tables. [file jcav12p0467s1.pdf]

Fig.S1 Details of the 41 OS patients

No.	Gender	Age	Tumor location	Surgical plan	Enneking stage	The follow-up	IRS
1	Female	15	Right distal femur	Tumor resection and prosthetic knee replacement	IIa	Lost follow-up in the 30th month	2.2
2	Female	21	Right distal femur	Hip disarticulation	IIIb	Lung metastasis/ Died in the 16th month	6.2
3	Male	18	Proximal right humerus	Tumor resection and prosthetic shoulder joint replacement	IIb	Tumor free	5.6
4	Male	21	Proximal left tibia	Tumor resection and prosthetic knee replacement	IIa	Lost follow-up in the 27th month	3.7
5	Female	30	Left distal tibia	Knee disarticulation	IIb	Lost follow-up in the 12th month	6.0
6	Male	28	Left distal femur	Tumor resection and autograft	IIIb	Brain metastasis/ Died in the 15th month	6.1
7	Male	67	Right distal femur	Hip disarticulation	IIIb	Lung metastasis/ Died in the 10th month	6.2
8	Female	13	Proximal left tibia	Tumor resection and prosthetic knee replacement	IIb	Tumor free	5.8
9	Female	16	Right distal femur	Tumor resection and prosthetic knee replacement	IIa	Tumor free	3.3
10	Female	22	Right distal femur	Tumor resection and prosthetic knee replacement	IIa	Tumor free	5.8
11	Male	19	Right distal radius	Elbow disarticulation	IIb	Tumor free	6.1
12	Female	17	Proximal left tibia	Tumor resection and prosthetic knee replacement	IIa	Tumor free	3.2
13	Female	21	Right distal femur	Hip disarticulation	IIb	Tumor free	4.1
14	Female	29	Proximal left humerus	Tumor resection and prosthetic shoulder joint replacement	IIa	Tumor free	4.3
15	Male	20	Right distal femur	Tumor resection and prosthetic knee replacement	IIa	Tumor free	4.1

16	Male	16	Left distal femur	Hip disarticulation	IIIb	Lung metastasis/ Died in the 14th month	5.2
17	Male	19	Left distal femur	Tumor resection and prosthetic knee replacement	IIb	Tumor free	4.4
18	Female	14	Right distal femur	Tumor resection and prosthetic knee replacement	IIb	Lung metastasis/ Died in the 30th month	5.9
19	Female	31	Proximal right femur	Hip disarticulation	IIIb	Lung metastasis/ Died in the 13th month	6.0
20	Female	29	Proximal right humerus	Tumor resection and prosthetic shoulder joint replacement	IIb	Tumor free	3.9
21	Male	36	Right distal femur	Tumor resection and prosthetic knee replacement	IIb	Lost follow-up in the 31st month	2.1
22	Female	19	Proximal left humerus	Tumor resection and prosthetic shoulder joint replacement	IIa	Tumor free	4.3
23	Female	16	Right distal femur	Tumor resection and autograft	IIa	Tumor free	3.3
24	Male	19	Left distal femur	Tumor resection and prosthetic knee replacement	IIb	Tumor free	3.0
25	Male	27	Right distal femur	Tumor resection and prosthetic knee replacement	IIa	Lost follow-up in the 29st month	2.8
26	Male	22	Proximal left tibia	Tumor resection and prosthetic knee replacement	IIb	Tumor free	3.0
27	Female	19	Left distal femur	Tumor resection and prosthetic knee replacement	IIIa	Brain metastasis/ Died in the 26th month	3.1
28	Male	18	Proximal left tibia	Tumor resection and prosthetic knee replacement	IIa	Tumor free	5.7
29	Female	69	Left distal tibia	Knee disarticulation	IIIa	Lung metastasis/ Died in the 32th month	5.1
30	Male	25	Right distal femur	Tumor resection and prosthetic knee replacement	IIa	Tumor free	4.5

31	Male	20	Left distal femur	Tumor resection and prosthetic knee replacement	IIb	Lung metastasis/ Died in the 30th month	4.3
32	Male	22	Proximal right tibia	Tumor resection and prosthetic knee replacement	IIa	Tumor free	5.7
33	Female	25	Right distal femur	Tumor resection and prosthetic knee replacement	IIa	Tumor free	3.5
34	Male	55	Right distal tibia	Knee disarticulation	IIIb	Lung metastasis/ Died in the 25th month	5.4
35	Male	16	Left distal femur	Tumor resection and prosthetic knee replacement	IIa	Tumor free	3.4
36	Female	17	Proximal left tibia	Tumor resection and prosthetic knee replacement	IIa	Tumor free	3.1
37	Male	23	Right distal femur	Tumor resection and prosthetic knee replacement	IIb	Lost follow-up in the 11st month	5.6
38	Male	19	Left distal femur	Tumor resection and prosthetic knee replacement	IIa	Tumor free	3.2
39	Female	21	Left distal femur	Tumor resection and prosthetic knee replacement	IIb	Lung metastasis/ Died in the 36th month	3.6
40	Male	19	Right distal femur	Tumor resection and autograft	IIb	Lost follow-up in the 26st month	5.7
41	Male	18	Proximal right tibia	Tumor resection and prosthetic knee replacement	IIa	Tumor free	3.6

Correlation with MUC15 expression and clinical characteristics

Clinical characteristics	n	MUC15	
		Expression level	p
Age≤24	29	4.35±0.23	0.48
Age＞24	12	4.66±0.40	
Male	22	4.52±0.27	0.68
Female	19	4.35±0.29	
Ennking stage(II)	33	4.21±0.21	0.01 (*)
Ennking stage(III)	8	5.41±0.37	
